# Supplementary material for: Correlations between distal sensorimotor polyneuropathy and cardiovascular complications in diabetic patients in the North-Eastern region of Hungary
Source: PLoS One. 2024 Jul 3;19(7):e0306482. doi: 10.1371/journal.pone.0306482 (PMC11221647; doi:10.1371/journal.pone.0306482)
Supplement: Attachment — Submitted filename: Response to Reviewers.docx [file pone.0306482.s002.docx]

*Marcelo Arruda Nakazone, M.D., Ph.D.*

*Academic Editor*

*PLOS ONE*

Dear Editor,

we have received the editorial response and the reviewers’ comments and questions regarding our revised manuscript titled “ Correlations between distal sensorimotor polyneuropathy and cardiovascular complications in diabetic patients in the North-Eastern region of Hungary" (PONE-D-24-06839). Thank you for the opportunity to submit our revised manuscript to your journal.

Please find below our point by point answers to the reviewers’ questions. Changes in the revised manuscript are marked on red. We also submit the final corrected version of the manuscript without markups.

**Responses to Editor:**

- We added this sentence to the *Acknowledgements* section: „The funders had no role in study design, data collection and analysis, decision to publish, or preparation of the manuscript.” (line 314-315, marked in blue)
- We confirm at this time that our submission contains all raw data required to replicate the results of your study, the values behind the means, standard deviations and other measures reported.
- ORCID iD for the corresponding author: https://orcid.org/0009-0005-6657-7579

**Responses to Reviewer #1:**

Thank you for the thoughtful review and the positive comments on our manuscript.

Responses to comments:

- The use of abbreviations has not been consistent throughout the text, either repeatedly spelled out again or not defined when it was first mentioned, e.g. DSPN, DN4. The authors ought to thoroughly cross check to keep the consistency.

Response: The Reviewer is absolutely correct, there were inconsistencies in the use of abbreviations in the manuscript. We have addressed this by making corrections and indicating the changes in red within the text. Unfortunately, the literature is also inconsistent in the usage of the terms "diabetic neuropathy" and "distal sensorimotor polyneuropathy" in diabetes. The most common cause of diabetic neuropathy is distal sensorimotor polyneuropathy (DSPN), however, diabetic autonomic neuropathy refers to damage to the autonomic nervous system. In clinical practice, DSPN is most commonly examined, and these data are often used when making statements regarding diabetic neuropathy. In our study, we examined the relationships of the DSPN, therefore, we used the abbreviation "DSPN" in the corrections and corrected the cases where we presented our own data or when distal sensorimotor neuropathy is mentioned in the referenced literature. When we talked about diabetic neuropathy in general, or when the literature referred to diabetic neuropathy, we used the terminology “diabetic neuropathy”.

- The whole third paragraph of Introduction is redundant.

Response: We corrected redundant information in the third paragraph of the „Introduction” section (line 62-65, marked in blue), resulting in the following sentences being included: „According to the international guidelines, it is recommended to assess neuropathic symptoms like paresthesia, burning sensations, and pinprick pain, along with changes in hot and cold sensation, during diabetic neuropathy screening. During the diagnostic process and the assessment of the severity of DSPN, symptoms and signs may be assessed through the utilization of questionnaire-based methods.” In the latter part of the paragraph, we had to incorporate sentences requested by Reviewer 3 (line 67-73, marked in blue), thus we were unable to significantly reduce this section of the introduction.

- The definition of diabetic neuropathy has not been described in details in Methods. Was the NCS done for each patient? or just based on the screening questionnaires? The scoring of the questionnaires also has to be defined for clearer justification.

Response: We added the definition of DSPN to the „Methods” section: „DSPN is characterized as an asymmetrical, length-dependent sensorimotor polyneuropathy attributable to metabolic and microvascular changes consequent to prolonged exposure to chronic hyperglycemia, compounded by cardiovascular risk factors” (line 122-124, marked in blue).

We also added these sentences to the „Methods” section: „A detailed assessment of DSPN was performed in all participants DN4 (Douleur Neuropathique 4 Questions) and Neuropathy Symptom Score questionnaires in screening for neuropathic pain syndrome, quantitative sensory testing, vibration perception threshold for the diagnosis of DSPN. Diabetic neuropathy was defined as scoring 3 points or higher on the Neuropathy Symptom Score or 4 points or higher on the DN4 questionnaire. Evaluation of superficial sensation is done with a monofilament test, vibration sensation with a tuning fork test, and instrumental assessment of vibration and temperature sensation thresholds may complement the examination (line 124-130, marked in blue).”

Objective assessment of neuropathic signs (vibration perception and sensory tests) and nerve conduction studies are reliable and reproducible in determining the extent and progression of neuropathy in diabetes. The evaluation of DSPN is based on typical symptoms and neuropathy screening tests. Therefore, in accordance with international recommendations, additional confirmatory electrophysiological testing of nerve conduction velocity (electroneurography) is typically unnecessary in diabetic patients. Electrophysiological tests or determination of intra-epidermal nerve fibre density during skin biopsy are only justified if other etiological factors arise or if the diagnosis is unclear due to atypical neurological symptoms (Davies 2006, Ziegler 2022). We added these sentences to the „Introduction” section (line 64-73, marked in blue)

References:

Tesfaye S, Boulton AJ, Dyck PJ, Freeman R, Horowitz M, Kempler P, et al. Diabetic neuropathies: update on definitions, diagnostic criteria, estimation of severity, and treatments. Diabetes Care. 2010;33(10):2285-93. doi: 10.2337/dc10-1303. PubMed PMID: 20876709; PubMed Central PMCID: PMCPMC2945176.

Ziegler D, Tesfaye S, Spallone V, Gurieva I, Al Kaabi J, Mankovsky B, et al. Screening, diagnosis and management of diabetic sensorimotor polyneuropathy in clinical practice: International expert consensus recommendations. Diabetes Res Clin Pract. 2022;186:109063. Epub 20210920. doi: 10.1016/j.diabres.2021.109063. PubMed PMID: 34547367.

Davies M, Brophy S, Williams R, Taylor A. The prevalence, severity, and impact of painful diabetic peripheral neuropathy in type 2 diabetes. Diabetes Care. 2006;29(7):1518-22. doi: 10.2337/dc05-2228. PubMed PMID: 16801572.

- Suggest to reduce the characteristic values to one decimal point, as the data presented is just too messy.

Response: We have made the requested changes (Table 1 and Table 2, marked in red).

- What is hepatopathy? Please define. Or use a more conventional terminology for the meant character.

Response: During the review of the documentation of our patients, we selected the data into a neuropathy register, in which, in view of the lack of clear documentation regarding the causes of liver disease, we formulated the term "hepatopathy" as a summary. 67 patients (10.8%) from the examined patient group suffered from liver disease, and during the reevaluation of the data, the causes of "hepatopathy" were identified as nonalcoholic fatty liver disease (NAFLD) and alcohol-induced liver damage. Accordingly, we added the meaning of hepatopathy in the „Method” section (line 119-120, marked in blue).

- Repeated typo of "acut" for "acute" in the tables.

Response: We have corrected the typos (Table 1 and Table 2, marked in red).

- Please remove the statistical test column in Table 2 as this has been defined in your Methods (Statistical Analysis).

Response: We have made the requested changes at the reviewer's request (Table 2).

- The data presented in Table 2 is just too messy - you can remove the "No" for each characteristics since this is not your concern.

Response: We have made the requested changes at the reviewer's request (Table 2).

- My major concern of the paper is how come the duration of the diabetes in not included in the multivariate logistic regression model? How did the authors include the variables into the model? This has not been clearly defined in the Methods as well.

Response: We find the Reviewer's comment entirely justified. Based on the reviewer's concern, we recalculated our multivariate logistic regression model to include duration of diabetes and re-edited "Table 3" accordingly. Although the duration of diabetes was significantly higher in patients with DSPN, we did not find an association between the duration of diabetes and the risk of DSPN in multivariate logistic regression analysis. We added this sentence to the „Discussion” section: „The risk of DSPN may increase with the duration of diabetes. Despite the significantly longer duration of diabetes in patients with DSPN, our multivariate logistic regression analysis did not reveal a significant association between duration of diabetes and the risk of DSPN.” (line 204-208, marked in blue)

- What is KORA? Again, please use and define your abbreviation appropriately.

Response: We have corrected the sentence in „Discussion” section (line 260-262, marked in blue)

- The discussion of 2-3 pages is just too long. I would suggest the authors to trim down the discussion.

Response: In accordance with the Reviewer's opinion, we significantly reduced the "Discussion" section, marking the deleted sentences within the manuscript. Accordingly, the literature references were reduced by 4 references in line with the deleted sentences, which was also indicated in the „References” section. However, we needed to consider the feedback from Reviewer 2 and Reviewer 3 regarding the inclusion of the Discussion and Limitations sections.

Thank you again for your thorough review of our manuscript and your valuable feedback!

**Responses to Reviewer #2:**

We would like to thank your valuable comments that improve our manuscript.

Responses to comments:

- The retrospective design could introduce recall bias or inaccuracies in medical records, potentially affecting the results' reliability. Due to its cross-sectional design, the study cannot identify risk factors for the development or progression of DSPN, only associations between DSPN and other vascular complications.

Response: The Reviewer is absolutely correct. In the retrospective design, we took into account recall bias and imprecision in the medical records, which could have potentially affected the reliability of our results. We added these sentences to the „Limitation” section: „because we identified cardiovascular and microvascular complications through outpatient and inpatient hospital records, it is important to acknowledge the potential for disease underreporting, as data from primary care sources are currently not fully available. Consequently, the correlations we found were not exploratory in nature.” (line 288-291, marked in blue), and „due to the retrospective nature of our study, any causal relationships should be approached with caution.” (line 299-300, marked in blue)

- While the study attempts to control for various factors, other potential confounders, such as lifestyle factors (diet, physical activity) and socioeconomic status, are not explicitly addressed.

Response: We find the Reviewer's comment entirely justified. We added this sentence to the „Discussion” section: „Due to gaps in the medical documentation, we were unable to explore many potential confounding factors in our study, such as lifestyle factors (diet, physical activity) and socioeconomic status.” (line 293-294, marked in blue)

- Abbreviations: please use abbreviations consistently (DSPN or DN).

Response: The Reviewer is absolutely correct, there were inconsistencies in the use of abbreviations in the manuscript. We have addressed this by making corrections and indicating the changes in red within the text. Unfortunately, the literature is also inconsistent in the usage of the terms "diabetic neuropathy" and "distal sensorimotor polyneuropathy" in diabetes. The most common cause of diabetic neuropathy is distal sensorimotor polyneuropathy (DSPN), however, diabetic autonomic neuropathy refers to damage to the autonomic nervous system. In clinical practice, DSPN is most commonly examined, and these data are often used when making statements regarding diabetic neuropathy. In our study, we examined the relationships of the DSPN, therefore, we used the abbreviation "DSPN" in the corrections and corrected the cases where we presented our own data or when distal sensorimotor neuropathy is mentioned in the referenced literature. When we talked about diabetic neuropathy in general, or when the literature referred to diabetic neuropathy, we used the terminology “diabetic neuropathy”.

- Patients’ selection: how did you exclude patients with heart failure other causes, like cardiomyopathies, atrial fibrillation or structural abnormalities?

Response: In the case of the patients selected for the examination, we were able to assess the comorbidities based on the available medical documentation, but we did not have the opportunity to organize detailed cardiology examinations. If we had cardiological findings, such as ECG, echocardiography, coronary CT results, etc., and etiological analysis of heart failure were also performed. However, reliable data were often insufficient for this purpose. Therefore, we did not investigate the etiology of heart failure, and the correlations we found were not of an exploratory nature, which we acknowledged in the "Limitation" section (line 288-291, marked in blue)

- You mentioned that confirmed DSPN (based on Toronto criteria) was a inclusion criteria, but you have a group without DSPN too. How did you define neuropathic signs? Could you provide nerve conduction data? You have used Neurometer to diagnose DSPN, but you did not show any data. You assessed neuropathic pain but did not provide any data about it.

Response: We added the definition of DSPN to the „Methods” section: „DSPN is characterized as an asymmetrical, length-dependent sensorimotor polyneuropathy attributable to metabolic and microvascular changes consequent to prolonged exposure to chronic hyperglycemia, compounded by cardiovascular risk factors (line 122-124, marked in blue). A detailed assessment of DSPN was performed in all participants DN4 (Douleur Neuropathique 4 Questions) and Neuropathy Symptom Score questionnaires in screening for neuropathic pain syndrome, quantitative sensory testing, vibration perception threshold for the diagnosis of DSPN. Diabetic neuropathy was defined as scoring 3 points or higher on the Neuropathy Symptom Score or 4 points or higher on the DN4 questionnaire. Evaluation of superficial sensation is done with a monofilament test, vibration sensation with a tuning fork test, and instrumental assessment of vibration and temperature sensation thresholds may complement the examination. The current perception threshold values measured with the Neurometer, as well as the DN4 and Neuropathy Symptom Score values, were utilized solely for establishing the diagnosis of neuropathy. However, we did not use the exact data in the present study, as we did not follow up the patients. „Objective assessment of neuropathic signs (vibration perception and sensory tests) and nerve conduction studies are reliable and reproducible in determining the extent and progression of neuropathy in diabetes. The evaluation of DSPN is based on typical symptoms and neuropathy screening tests. Therefore, in accordance with international recommendations, additional confirmatory electrophysiological testing of nerve conduction velocity (electroneurography) is typically unnecessary in diabetic patients. Electrophysiological tests or determination of intra-epidermal nerve fibre density during skin biopsy are only justified if other etiological factors arise or if the diagnosis is unclear due to atypical neurological symptoms.” We added these sentences to the „Introduction” section (line 65-73, marked in blue)

References:

Tesfaye S, Boulton AJ, Dyck PJ, Freeman R, Horowitz M, Kempler P, et al. Diabetic neuropathies: update on definitions, diagnostic criteria, estimation of severity, and treatments. Diabetes Care. 2010;33(10):2285-93. doi: 10.2337/dc10-1303. PubMed PMID: 20876709; PubMed Central PMCID: PMCPMC2945176.

Ziegler D, Tesfaye S, Spallone V, Gurieva I, Al Kaabi J, Mankovsky B, et al. Screening, diagnosis and management of diabetic sensorimotor polyneuropathy in clinical practice: International expert consensus recommendations. Diabetes Res Clin Pract. 2022;186:109063. Epub 20210920. doi: 10.1016/j.diabres.2021.109063. PubMed PMID: 34547367.

Davies M, Brophy S, Williams R, Taylor A. The prevalence, severity, and impact of painful diabetic peripheral neuropathy in type 2 diabetes. Diabetes Care. 2006;29(7):1518-22. doi: 10.2337/dc05-2228. PubMed PMID: 16801572.

- In the table of baseline characteristic, you show hepatopathy. How did you define hepatopathy? Does it mean NAFLD or NASH?

Response: During the review of the documentation of our patients, we selected the data into a neuropathy register, in which, in view of the lack of clear documentation regarding the causes of liver disease, we formulated the term "hepatopathy" as a summary. 67 patients (10.8%) from the examined patient group suffered from liver disease, and during the reevaluation of the data, the causes of "hepatopathy" were identified as nonalcoholic fatty liver disease (NAFLD) and alcohol-induced liver damage. Accordingly, we added the meaning of hepatopathy in the „Method” section (line 119-120, marked in blue).

- The association between DSPN and CVD is a clinically important result. However, regardless of the results obtained from the regression model, it is important to emphasize that accurate predictions cannot be guaranteed by cross sectional study. Please mention it in a limitation section.

Response: The Reviewer is absolutely correct, we modified the "Limitation" section as follows: „because we identified cardiovascular and microvascular complications through outpatient and inpatient hospital records, it is important to acknowledge the potential for disease underreporting, as data from primary care sources are currently not fully available. Consequently, the correlations we found were not exploratory in nature.” (line 288-291, marked in blue)

- It's a cross-sectional analysis, so you only have associations with the prevalence of DSPN but no data on the risk of DSPN.

Response: We agree with the Reviewer, we added the following sentence to the "Limitation" section: „Finally, due to the retrospective nature of our study, any causal relationships should be approached with caution.” (line 299-300, marked in blue)

- There are only few studies showing association between peripheral neuropathy and cardiovascular risk. However, a study with 30-month follow-up period could show that PN is associated with increased risk for a first cardiovascular event among individuals with type 2 diabetes. Neuropathy was nevertheless only diagnosed with a neurofilament test. Heart. 2014 Dec;100(23):1837-43.

Response: Thank you for the Reviewer's comment; we fully agree with the reviewer's addition. We added the following sentence to the "Discussion" section: „Previous studies have confirmed the increased risk of diabetic neuropathy in individuals with diabetes who have survived myocardial infarction, additionally, DSPN has been demonstrated to correlate with an increased risk of initial cardiovascular events in T2DM” (line 260-262, marked in blue). We have also added the recommended literature reference to the "References" section (Ref 39).

- Line 210: Effective management of diabetes can decelerate the progression of DSPN in individuals with Type 1 Diabetes (T1D), yet it only yields limited benefits for those with Type 2 Diabetes (T2D) (Cochrane Database Syst Rev. 2012;6(6):CD007543.).

Response: The Reviewer is absolutely correct, we modified the " Discussion" section as follows: „Effective management of diabetes may reduce the progression of DSPN in individuals with T1DM, but it offers only limited benefits for those with T2DM” (line 215-216, marked in blue). We have also added the recommended literature reference to the "References" section (Ref 28).

- Line 257: the connection btw CAN and CVD is well known, but you did not provide any data on autonomic neuropathy. It is also a limitation of the study.

Response: We agree with the Reviewer, we added the following sentence to the "limitation" section: „The relationship between cardiac autonomic neuropathy and cardiovascular diseases is well known, but we did not have any data regarding autonomic neuropathy in the medical records” (line 296-297, marked in blue).

Thank you again for your thorough review of our manuscript and your valuable feedback!

**Reviewer #3:**

Thank you for your review and constructive comments on our manuscript.

Responses to comments:

- it is a retrospective study

Response: The Reviewer is absolutely correct. In the retrospective design, we took into account recall bias and imprecision in the medical records, which could have potentially affected the reliability of our results. We added these sentences to the „Limitation” section: „because we identified cardiovascular and microvascular complications through outpatient and inpatient hospital records, it is important to acknowledge the potential for disease underreporting, as data from primary care sources are currently not fully available. Consequently, the correlations we found were not exploratory in nature.” (line 288-291, marked in blue), and „due to the retrospective nature of our study, any causal relationships should be approached with caution.” (line 299-300, marked in blue)

- high number of exclusions, criteria for exclusion are not clearly stated i.e. what is the difference between “without any detailed electronic medical records” and “incomplete electronic health documentation”.

Response: We consider the Reviewer's comment to be valid, we modified the " Enrollment of study participants" section as follows: „During our investigation, we assessed a total of 1237 diabetic patients who underwent neuropathy screening at the Diabetic Neuropathy Center, Department of Internal Medicine, Faculty of Medicine, University of Debrecen, between January 1, 2017 and December 31, 2021. We excluded patients in whom other causes of polyneuropathy were identified (n=467), such as chronic alcohol consumption, hematological disorders, chemotherapy, malignant diseases, autoimmune musculoskeletal diseases, or vitamin B12 deficiency. The inclusion criterion was the availability of hospital treatment records for the patients, so those without detailed electronic medical records were excluded (n=102). Among the remaining patients, where confirmation of distal sensorimotor polyneuropathy was obtained, patients with incomplete data corresponding to baseline characteristics were excluded (n=47). Study design flowchart of diabetic patients with DSPN are depicted in Fig 1.” (line 91-99, marked in blue). Figure 1 has also been modified for clarity of interpretation.

- how was neuropathy of non-diabetic origin defined. Diabetes at diagnosis is not the same as the time living with diabetes as it is often asymptomatic.

Response: We added the definition of DSPN to the „Methods” section: „DSPN is characterized as an asymmetrical, length-dependent sensorimotor polyneuropathy attributable to metabolic and microvascular changes consequent to prolonged exposure to chronic hyperglycemia, compounded by cardiovascular risk factors. A detailed assessment of DSPN was performed in all participants DN4 (Douleur Neuropathique 4 Questions) and Neuropathy Symptom Score questionnaires in screening for neuropathic pain syndrome, quantitative sensory testing, vibration perception threshold for the diagnosis of DSPN. Diabetic neuropathy was defined as scoring 3 points or higher on the Neuropathy Symptom Score or 4 points or higher on the DN4 questionnaire. Evaluation of superficial sensation is done with a monofilament test, vibration sensation with a tuning fork test, and instrumental assessment of vibration and temperature sensation thresholds may complement the examination” (line 122-130, marked in blue)

References:

Tesfaye S, Boulton AJ, Dyck PJ, Freeman R, Horowitz M, Kempler P, et al. Diabetic neuropathies: update on definitions, diagnostic criteria, estimation of severity, and treatments. Diabetes Care. 2010;33(10):2285-93. doi: 10.2337/dc10-1303. PubMed PMID: 20876709; PubMed Central PMCID: PMCPMC2945176.

Ziegler D, Tesfaye S, Spallone V, Gurieva I, Al Kaabi J, Mankovsky B, et al. Screening, diagnosis and management of diabetic sensorimotor polyneuropathy in clinical practice: International expert consensus recommendations. Diabetes Res Clin Pract. 2022;186:109063. Epub 20210920. doi: 10.1016/j.diabres.2021.109063. PubMed PMID: 34547367.

- the criteria for inclusion are not clearly stated (apart from the reference to the Toronto criteria).

Response: We agree with the Reviewer, the inclusion criteria were not clearly stated and the diagnosis of neuropathy also needed to be supplemented. The diagnosis of DSPN was detailed as follows in the „Methods” section: „A detailed assessment of DSPN was performed in all participants DN4 (Douleur Neuropathique 4 Questions) and Neuropathy Symptom Score questionnaires in screening for neuropathic pain syndrome, quantitative sensory testing, vibration perception threshold for the diagnosis of DSPN. Diabetic neuropathy was defined as scoring 3 points or higher on the Neuropathy Symptom Score or 4 points or higher on the DN4 questionnaire. Evaluation of superficial sensation is done with a monofilament test, vibration sensation with a tuning fork test, and instrumental assessment of vibration and temperature sensation thresholds may complement the examination.” „The diagnosis of DSPN was established based on the presence of neuropathic symptoms or signs recorded according to neuropathy questionnaires, as well as the presence of at least one abnormal result in quantitative sensory testing, vibration perception threshold, or current perception threshold tests, in accordance with international guidelines.” (line 124-130 and line 133-137, marked in blue)

- the data for the detailed clinicals assessments are not shown including the neurological clinical examination, no mention of a standardised and validated measures of neuropathy such as nerve conduction studies or skin biopsy for intra-epidermal nerve fibre density.

Response: We accept the Reviewer's concerns, but objective assessment of neuropathic signs (vibration perception and sensory tests) and semiquantitative nerve conduction studies (like current perception threshold test with a Neurometer) are reliable and reproducible in determining the extent and progression of neuropathy in diabetes (Tesfaye 2010, Ziegler 2022). The evaluation of DSPN is based on typical symptoms and neuropathy screening tests. Therefore, in accordance with international recommendations, additional detailed neurological clinical examination and confirmatory electrophysiological testing of nerve conduction velocity (electroneurography) are typically unnecessary in diabetic patients. Electrophysiological tests or determination of intra-epidermal nerve fibre density during skin biopsy are only justified if other etiological factors arise or if the diagnosis is unclear due to atypical neurological symptoms (Davies 2006, Ziegler 2022). We added these sentences to the „Introduction” section (line 62-73, marked in blue).

References:

Tesfaye S, Boulton AJ, Dyck PJ, Freeman R, Horowitz M, Kempler P, et al. Diabetic neuropathies: update on definitions, diagnostic criteria, estimation of severity, and treatments. Diabetes Care. 2010;33(10):2285-93. doi: 10.2337/dc10-1303. PubMed PMID: 20876709; PubMed Central PMCID: PMCPMC2945176.

Ziegler D, Tesfaye S, Spallone V, Gurieva I, Al Kaabi J, Mankovsky B, et al. Screening, diagnosis and management of diabetic sensorimotor polyneuropathy in clinical practice: International expert consensus recommendations. Diabetes Res Clin Pract. 2022;186:109063. Epub 20210920. doi: 10.1016/j.diabres.2021.109063. PubMed PMID: 34547367.

Davies M, Brophy S, Williams R, Taylor A. The prevalence, severity, and impact of painful diabetic peripheral neuropathy in type 2 diabetes. Diabetes Care. 2006;29(7):1518-22. doi: 10.2337/dc05-2228. PubMed PMID: 16801572.

- current perception threshold testing using the Neurometer is not an accepted measure of neuropathy.

Response: We completely agree with the Reviewer, we added the modification to the *Methods* section: „We also assessed the function of peripheral sensory nerves using a current perception threshold test with a Neurometer® (Neurotron Inc., Baltimore, Maryland, USA, 2002). This neurodiagnostic device has been previously reported for detecting DSPN; however, it is not suitable as a standalone tool for diagnosing diabetic neuropathy” (line 130-133, marked in blue). We did not only use the results of the Neurometer for the diagnosis of DSPN, but evaluated the neuropathic symptoms based on the neuropathy questionnaires, and the quantitative sensory test or the vibration perception threshold test also had to be abnormal when evaluating the results of the current perception threshold test determined with the Neurometer.

- multiple logistic regression model lacks detail on model fit statistics, the estimated coefficients for each independent variable included in the model, confounding variables and mention of assumptions.

Response: The estimated coefficients for each independent variable were included in the model, we have updated the model as Reviewer 1 requested (adding duration of diabetes). We can present unadjested point estimations as well (odds ratio/coefficient) but we used instead simple analysis (chi squared and wilcoxon ranksum test) for more informative tables (Table 2). Potential influencing variables are already included in the model based on the simple analysis (Table 2) moreover general confounders also included (like age and gender).

Regarding model fitting, the pseudo R-squared value (0.2201), while not exceptionally high, indicates a moderate explanatory power of the model relative to the complexity of biological data. Both Akaike’s information criterion (582.337) and Bayesian information criterion (630.739) values provided suggest that the model is reasonably good.

Thank you again for your thorough review of our manuscript and your valuable feedback!
