# Supplementary material for: Correlations between distal sensorimotor polyneuropathy and cardiovascular complications in diabetic patients in the North-Eastern region of Hungary
Source: PLoS One. 2024 Jul 3;19(7):e0306482. doi: 10.1371/journal.pone.0306482 (PMC11221647; doi:10.1371/journal.pone.0306482)
Supplement: Attachment — Submitted filename: Response to Reviewers2.docx [file pone.0306482.s003.docx]

*Marcelo Arruda Nakazone, M.D., Ph.D.*

*Academic Editor*

*PLOS ONE*

Dear Editor,

We have received the editor's response as well as the reviewer's comments and questions regarding our revised manuscript entitled "Relationships between distal sensorimotor polyneuropathy and cardiovascular complications in diabetic patients in the North-Eastern Hungarian region" (PONE-D-24-06839). Thank you for the opportunity to resubmit our revised manuscript to your journal.

Below you will find our point-by-point answers to the reviewer's questions. Changes to the revised manuscript were made in proofreading and marked in yellow. We also submit the final corrected version of the manuscript without markings.

**Review Comments to the Author**

**Reviewer #1:** The manuscript has improved after the correction, but I still have several comments to make.

Page 3 Line 62: You mean reduce the complications caused by diabetes or diabetic neuropathy? Please clarify.

Response: The Reviewer is absolutely right, we have corrected the sentence: „Early recognition of DSPN, modification of risk factors, appropriate glycaemic control, blood pressure control, maintenance of body weight, regular physical activity, treatment of hypertension and abnormal blood lipid levels can reduce the development of complications caused by diabetes mellitus.” (line 98)

Page 3 Line 70-74: Inappropriately elaborated here. This should be in your limitation to justify since you did not perform the tests.

Response: We added the following sentence to the "Limitation" section: „Sixth, as part of the diagnosis of neuropathy, we did not perform an electrophysiological examination (electroneurography) or determine the intraepidermal nerve fiber density from a skin biopsy.” (line 404-405)

The authors do not seem to understand the comments from multiple reviewers when we questioned how did they define their cohort of diabetic neuropathy. Page 5 Line 123-125 is redundant.

Response: In accordance with the Reviewer's request, the unnecessary sentence was deleted, and our group of patients with diabetic neuropathy was reworded as follows: „In our study, we selected diabetic patients in whom the typical symptoms of DSPN were observed, typically numbness, tingling, burning pain, sensitivity to touch, muscle weakness, and coordination disorders.” (line 160-162)

Page 5 Line 125-128: Grammarly incorrect. Please rephrase the whole sentence.

Response: Agreeing with the Reviewer's opinion, we rephrased the sentence: „A detailed evaluation of DSPN was performed in all participants using the DN4 (Douleur Neuropathique 4 Questions) and Neuropathy Symptom Score questionnaires during the screening of neuropathic pain.” (line 162-163)

Page 5 Line 133-134: This is redundant since you have justified using other tests plus Neurometer to define the neuropathy.

Response: In accordance with the Reviewer's request, the unnecessary sentence was deleted, and we rephrased the sentence: „The superficial sensation was evaluated with a monofilament test, the vibration detection with a tuning fork test, and the examination was completed by an instrumental evaluation of the temperature detection thresholds.” (line 165-167)

Significant discrepancies in the values presented in text (including abstract) and tables. Please align all the values, including decimal points.

Response: Thank you very much for the Reviewer's comment, we did not follow the multiple changes in the tables during the reviewer process exactly in the text, we modified these discrepancies in the "Abstract" and the "Results" (line 27-28, line 35-38, line 192, line 212-214, line 216, line 243-246)

Page 8 Line 177 and Abstract: HbA1c above 7% - Is this variable continuous or categorical when included in the model? If this is categorical, this has not been clearly defined in Methods.

Response: The parameter HbA1c above 7% is included as a continuous variable in the analysis, as is the parameter albumin/creatinine ratio above 3 mmol/mol. The HbA1c cut-off value set at 7% is arbitrary but adapted to the health insurance conditions in Hungary. This is because the Hungarian health funder considers a carbohydrate intake above this value to be insufficient for diabetics and allows for the funded prescription of modern antidiabetic medications. In contrast, international guidelines consider an HbA1c value between 6-8% acceptable, taking into account individual preferences, age, comorbidities, duration of diabetes, and other factors.

How did the authors decide which variables to be included in the multivariate logistic regression model? This is not clear from the statistical analysis. And, could that be the reason why they did not find the duration of diabetes to be significant? The significance of independent predictors is very much depended on the variables that you include in the model.

Response: In a multiple logistic regression model, the selected variables included parameters for carbohydrate homeostasis (HbA1c, duration of diabetes), microvascular complications (eGFR, albumin/creatinine ratio, diabetic retinopathy), and lifestyle and macrovascular complications (composite cardiovascular endpoint, high blood pressure, smoking). We have added the mentioned sentence in "Statistical analysis”. (line 183-187) We tested several multiple logistic regression models to examine the influence of diabetes duration, but in none of them did diabetes duration prove to be a significant predictor of DSPN.

Page 9 Paragraph 3: In the end if the authors still did not find the duration of diabetes to be significant in multivariate analysis, please explain. This was not explained in the discussion.

Response: We hypothesize that, despite the statistical difference, both patient groups can be considered long-standing diabetics, which may explain why the duration of diabetes did not prove to be a significant predictor of DSPN in the multivariable logistic regression model. Accordingly, we have added the mentioned sentence in "Discussion". (line 310-312)

Page 9 Paragraph 4: But your cohort significantly contains more T2DM than T1DM, how do you explain this?

Response: The statements in the "Discussion" section refer to the association between poor glycemic control and the risk of diabetic neuropathy in both type 1 and type 2 diabetes mellitus. However, effective treatment of diabetes mainly reduces the progression of DSPN in patients with T1DM, and for those with T2DM, glycemic control has only limited benefits. The results of our study are consistent with previous findings that hyperglycemia may be significantly associated with an increased risk of diabetic neuropathy. Accordingly, we have modified the mentioned section in "Discussion". (line 317-320)

Page 11 Line 266-267: How would it be your study implications when you did not actually perform the tests?

Response: The Reviewer is absolutely right, we have corrected the sentence: „Consenquently, the use of diabetic neuropathy screening tests may reveal peripheral nerve dysfunction in recently diagnosed diabetic patients, enabling more appropriate glycemic control and increased monitoring of other microvascular complications during diabetes care.” (line 378-383)

Thank you again for your thorough review of our manuscript and your valuable feedback!

**Reviewer #2: (No Response)**

Thank you again for reviewing our manuscript and supporting its acceptance!
